# Optimal control of Typhoid fever transmission under environmental and public health interventions

**DOI:** 10.1371/journal.pone.0351747

**Published:** 2026-06-24

**Authors:** John Amoah-Mensah, Mohamedahmed Mirghani Hassan Mohamed, Reindorf Nartey Borkor, Rhoda Afutu, Nicholas Kwasi-Do Ohene Opoku

**Affiliations:** 1 Department of Computer Science, Sunyani Technical University, Sunyani, Ghana; 2 Department of Mathematical Sciences, African Institute for Mathematical Sciences, Accra, Ghana; 3 Department of Mathematics, Kwame Nkrumah University of Science and Technology, Kumasi, Ghana; 4 Department of Mathematics, University of Cape Coast, Cape Coast, Ghana; Stellenbosch University, SOUTH AFRICA

## Abstract

**Background:**

This study investigates the transmission dynamics of typhoid fever and assesses the impact of environmental factors and public health interventions on disease spread. Typhoid fever, caused by Salmonella Typhi, remains a major public health concern in regions with poor sanitation, high population density, and limited access to clean water. Although environmental contamination plays a critical role in sustaining transmission, its contribution is often under explored in mathematical modeling studies.

**Methods:**

We developed a deterministic compartmental model incorporating environmental transmission pathways to better understand the role of contaminated water sources and human-environment interactions in the spread of typhoid fever. The model is formulated as a system of nonlinear ordinary differential equations. The basic reproduction number, *R*_0_ was derived using the next-generation matrix approach to determine the threshold conditions for disease persistence. We analyzed the existence and stability of the disease-free and endemic equilibrium points, establishing local and global stability results for $R_{0}\leq 1$ and *R*_0_ > 1, respectively. Sensitivity analysis on the reproduction number and the endemic equilibrium was conducted to identify parameters with the greatest influence on disease transmission. Furthermore, the model was extended to an optimal control framework incorporating two intervention strategies: public health education campaigns and treatment of contaminated water bodies. Pontryagin’s Maximum Principle was applied to characterize the optimal controls and derive the associated optimality system. Model parameters were estimated using reported typhoid fever data from Ethiopia obtained through the World Health Organization. Numerical simulations were performed to evaluate the impact of individual and combined intervention strategies.

**Results:**

Simulation results indicate that the combined implementation of environmental sanitation measures and educational interventions significantly reduces disease burden, particularly during outbreak periods.

**Conclusion:**

These findings highlight the importance of integrating environmental management and community-based public health strategies in typhoid control programs.

## Introduction

Typhoid fever is an infectious febrile disease caused by the bacterium *Salmonella typhi* [1]. The disease is strongly associated with inadequate sanitation, overcrowding, and informal settlements, where environmental conditions facilitate bacterial persistence and transmission [1]. It occurs in both rural and urban settings lacking reliable access to safe water and proper waste disposal systems. Transmission primarily occurs through ingestion of food or water contaminated with fecal material from infected individuals [2]. The clinical presentation typically includes prolonged fever, abdominal pain, malaise, headache, nausea, constipation and diarrhea, and general weakness [3]. In some cases, patients may develop a rash, and severe untreated infections can lead to life-threatening complications [4]. Symptoms may persist for several weeks. Importantly, asymptomatic individuals can shed the bacteria and contribute to continued transmission within the population [5]. Without appropriate treatment, mortality rates have been reported to reach approximately 10% [3].

Typhoid fever is estimated to cause between eleven and twenty-one million cases and more than one hundred thousand of deaths annually [3]. It continues to represent a significant public health burden, particularly in low- and middle-income countries (LMICs) [3]. The majority of endemic regions are located in Southeast Asia and sub-Saharan Africa, which collectively account for a substantial proportion of the global morbidity and mortality. In these regions, poor sanitation and inadequate access to clean water are major contributors to the continued prevalence of typhoid and paratyphoid fever [3]. Although substantial progress has been made in managing typhoid fever, including the use of antibiotics and the development of several vaccines such as Typhoid Conjugate Vaccine (TCV), Typhoid Vi capsular polysaccharides vaccine, and Ty21a vaccine, the disease remains endemic in these regions [6]. According to [7] the persistent challenge lies in the insufficient focus on improving environmental sanitation, which is a critical determinant for the transmission of this disease. A crucial call has been the need for a multi-faceted approach that integrates water, sanitation, and hygiene (WASH) activities to reduce the incidence of Typhoid fever.

Mathematical models have been among the approaches implemented to understand the transmission dynamics of typhoid fever. However, most of these models have primarily focused on transmission dynamics [8–12], while others have investigated the effectiveness of various control strategies, particularly those centered on vaccination, treatment, or a combination of both [13–20]. Unfortunately, incorporating environmental factors into typhoid fever models has not received much attention, with notable exceptions including the works of [17,19,20]. This highlights an important gap in the current body of research, suggesting that the influence of environmental factors remains under explored in the context of typhoid fever control.

For example, Tilahun et al. [19] developed an SCIR-B compartmental model to analyze typhoid fever outbreaks by incorporating three time-dependent controls: prevention, treatment, and screening. They argued that the eradication of typhoid requires a combination of prevention and treatment mechanisms, with screening playing a lesser role. However, we argue that without a well-defined prevention strategy, stakeholders may struggle to identify effective approaches for interrupting transmission. A similar assessment was made by Abboubakar et al. [20], whose model focused on vaccination, individual protection, and treatment. However, without a proper environmental sanitation approach, it becomes challenging to assess the measures needed for controlling the disease. The study by Khan et al. [21] focused on the dynamical behavior of a nonlinear typhoid model, employing numerical methods such as the NSFD scheme for stability analysis. Their approach, however, mainly emphasized the dynamical properties of the disease without incorporating key environmental factors. Similarly, Dayan et al. [22] introduced a model with fuzzy parameters to address uncertainties in infection protection but largely overlooked the role of environmental bacterial persistence in water sources, which is a critical pathway for typhoid transmission.

In a different development, Lawal et al. [23] introduced a medically hygienic compartment in their model, focusing on personal hygiene as a control strategy. While their model underscores the importance of hygienic practices, it did not explicitly pay attention to key environmental factors such as bacterial survival and the impact of water treatment, which are central to typhoid fever transmission. To address some of the questions raised in [23], Sharma et al. [24] incorporated incidence rates and saturated treatment strategies, showing that nonlinear dynamics in treatment capacity significantly influence disease control. Although their model highlights the importance of saturated treatment, it failed to establish key bacterial growth thresholds needed to serve as a baseline for water treatment interventions. The study by Nana-Kyere et al. [25] provided insight into the costs involved in controlling typhoid fever transmission by applying cost-effectiveness analysis to assess various control strategies. Their study highlighted the economic impact of vaccination and personal protection. While the study is valuable for resource allocation, the cost of treatment was not assessed. We observed that most of these models focus primarily on human-to-human transmission and treat environmental bacteria as a static or overly simplified compartment. However, this does not provide a realistic representation of disease dynamics. Hence, we propose a novel approach by representing environmental bacterial dynamics using a logistic growth model. This allows us to capture the long-term persistence of bacteria in the environment and to model the impact of environmental interventions more accurately. Consequently, the model introduces an additional layer of ecological realism, making it distinct from many existing models. In modeling human-to-human transmission, we incorporate a saturated incidence rate to account for crowding effects, thereby providing a more realistic representation of disease transmission in high-density urban settings and low-resource environments. Furthermore, while existing models often focus on isolated control measures such as vaccination or treatment, our model integrates environmental and behavioral factors by emphasizing the roles of water treatment and educational campaigns. This approach addresses environmental transmission pathways that are frequently overlooked in previous studies while also providing a comprehensive framework particularly relevant for endemic regions where waterborne transmission remains a major concern.

## Materials and methods

### Model formulation

**Fig 1:**
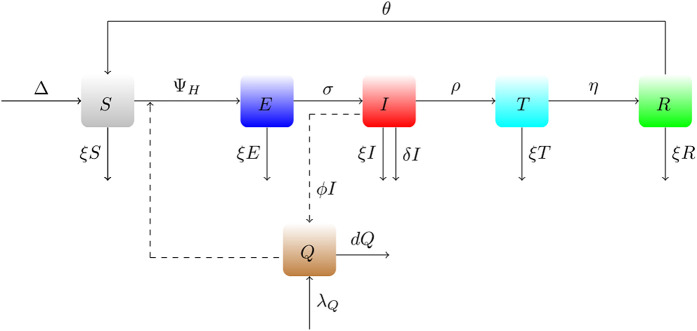
Transmission dynamics of typhoid fever in an environmental settings.

Typhoid disease transmission is inherently ecological and social. Thus, human hosts and environmental reservoirs are both central to understanding its spread [26]. Therefore, our model integrates both human transmission mechanisms and environmental bacterial dynamics within a single system. At time *t*, the total human population, denoted by *N*(*t*), is structured into susceptible (*S*), exposed (*E*), infected (*I*), treated (*T*), and recovered (*R*) compartments. The environmental load of *Salmonella Typhi* bacteria is denoted by *Q*, representing bacterial contamination in water and food sources. The dynamics and structure of disease transmission are shown in Fig 1.

At a rate Δ, the susceptible population increases through birth or immigration, after which individuals may become exposed through the force of infection given in Eq (1).


$$\begin{array}{c} \Psi_{H}(t)=\frac{\beta_{Q}Q}{Q+\alpha_{0}}+\frac{\beta_{H}I}{1+\alpha_{1}I}. \end{array}$$ (1)

The force of infection reflects two principal transmission pathways: infection through contact with infected humans and infection through environmental contamination. From Eq (1), the parameter $\beta_{H}$ denotes the transmission rate due to contact with infected individuals, while $\beta_{Q}$ captures environmental transmission, where susceptible individuals ingest bacteria from contaminated water or other environmental reservoirs. This route is particularly important in settings with poor sanitation and unsafe water supply, conditions identified by the WHO as major risk factors for typhoid transmission [24]. The terms $\alpha_{0}$ and $\alpha_{1}$ represent saturation effects. Specifically, $\alpha_{0}$ prevents unbounded infection rates at high environmental bacterial densities, reflecting the ecological principle that increases in *Q* do not necessarily result in proportional increases in transmission. Similarly, $\alpha_{1}$ reflects saturated human contact, whereby the effective transmission rate per infected individual decreases as the number of infectious contacts increases, thereby accounting for behavioral changes and contact limitations at high prevalence.

Afterwards, individuals progress from the exposed class to the infectious class at rate σ. A proportion of infected individuals die from typhoid fever at rate δ, reflecting disease severity and case fatality [26]. Infected individuals receive antibiotic treatment at rate ρ, moving into the treated compartment, and subsequently recover at rate η. Recovered individuals may lose immune protection at rate θ, consistent with WHO observations that immunity following infection or vaccination does not always confer lifelong protection. All human compartments are subject to a natural mortality rate of ξ.

Unlike models that treat the environmental reservoir as static, we explicitly model the growth and persistence of *Salmonella Typhi* in the environment using a logistic growth term


$$\begin{array}{c} \lambda_{Q}=zQ\left(1-\frac{Q}{C}\right), \end{array}$$

where *z* is the intrinsic growth rate of the bacteria and *C* denotes the carrying capacity of the environment. Infected individuals shed bacteria into the environment at rate ϕ, for example through fecal contamination of water systems, while bacteria decay at rate *d* due to natural environmental stressors.

Mathematically, Fig 1 can be written in six systems of differential equations as


$$\begin{array}{c} \left\{ \begin{array}{cc} \frac{dS}{dt} & =\Delta +\theta R-\Psi_{H}S-\xi S,\\ \frac{dE}{dt} & =\Psi_{H}S-(\xi +\sigma )E,\\ \frac{dI}{dt} & =\sigma E-(\xi +\delta +\rho )I,\\ \frac{dT}{dt} & =\rho I-(\xi +\eta )T,\\ \frac{dR}{dt} & =\eta T-(\xi +\theta )R,\\ \frac{dQ}{dt} & =\lambda_{Q}+\phi I-dQ. \end{array}\right. \end{array}$$ (2)

System (2) is qualitatively analyse with the results provided in the S1 File.

### Parameter estimation

**Fig 2:**
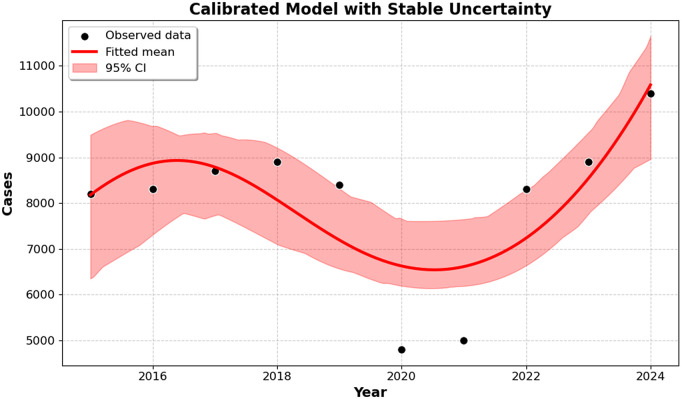
Observed data and model fit for typhoid fever incidence.

In this subsection, the parameters of system 6 are estimated to examine the trend of typhoid fever incidence. The model was fitted to reported annual typhoid cases from 2015 to 2024. The dataset used is aggregated, anonymized, and publicly accessible; hence, no ethical approval was required. The model incidence was defined as


$$\begin{array}{c} ℐ(t)=k\;\sigma E(t), \end{array}$$ (3)

where *k* accounts for possible under-reporting. Unknown parameters were estimated using a nonlinear least squares approach by [27] minimizing the difference between observed data and model predictions. The objective function is given by


$$\begin{array}{c} \min_{\Theta }\sum_{i=1}^{n}{\left[X_{r}(t_{i})-Y(t_{i},\Theta )\right]}^{2}, \end{array}$$ (4)

where $X_{r}(t_{i})$ represents the reported typhoid cases and $Y(t_{i},\Theta )=k\;\sigma E(t_{i};\Theta )$ denotes the corresponding model output with parameter set Θ. System 6 was solved numerically, and the optimization procedure was implemented in Python using standard techniques. The estimated parameter values are presented in Table 1, while the corresponding model fit is shown in Fig 2. Observed data and model fit for typhoid fever incidence. To further assess the robustness of estimated parameters, a local approximation of the covariance matrix was constructed from the Jacobian of the residuals at the optimal solution. We assumed an approximate normality of the estimator, and define the covariance matrix as $Cov(\Theta )\approx \omega^{2}(J^{T}J{)}^{-1},$, where *J* is the Jacobian matrix and $\omega^{2}$ is the residual variance. Using this covariance structure, 500 Monte Carlo samples of the parameter vector were generated under a multivariate normal assumption. The resulting ensemble of simulations was used to construct 95% confidence intervals for the model output, providing a measure of uncertainty around the fitted trajectory. Fig 2 depicts the epidemic trend and reproduces the observed multi-year wave pattern.

**Table 1:** Parameter values for Typhoid fever model.

Parameter	Value	Reference
Δ	4990	Fixed
ξ	0.0195	Estimated
*d*	0.1	[28]
$\beta_{Q}$	0.003	[19]
$\beta_{H}$	0.00097	Estimated
ϕ	0.026	[28]
δ	0.06	Estimated
ρ	0.551	Estimated
η	0.03	Estimated
*z*	0.01	[29]
σ	0.051	Estimated
θ	0.5	Estimated
$\alpha_{0}$	0.5	Estimated
$\alpha_{1}$	0.7	Estimated
*C*	5000	[28]

The bootstrap-based 95% confidence interval indicates moderate parameter uncertainty, particularly at the temporal boundaries where data constraints are weaker.

### Sensitivity assessment of reproduction number

A sensitivity analysis is conducted to assess the influence of each parameter on the transmission dynamics of Typhoid fever. Applying Eq (5), we obtain the results shown in Table 2.


$$\begin{array}{c} U_{p}^{R_{0}}=\frac{\partial R_{0}}{\partial p}\frac{p}{R_{0}}. \end{array}$$ (5)

**Fig 3:**
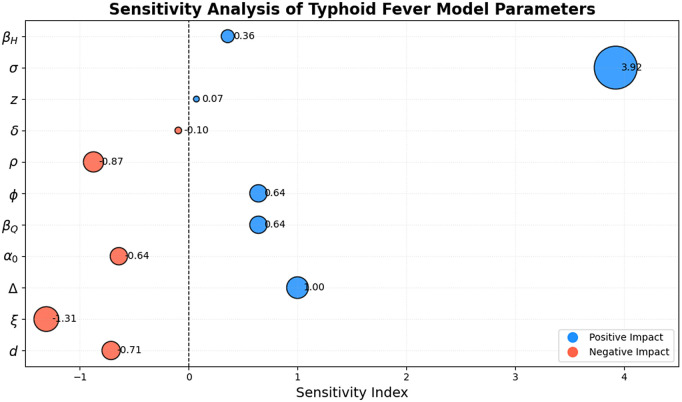
Graph of sensitivity indices with respect to parameters in the *R*_0_.

The sensitivity analysis in Table 2 and Fig 3 reveals important biological implications for the transmission dynamics of typhoid fever.

**Table 2:** Sensitivity indices of model parameters and their impact on Typhoid disease transmission.

Parameter	Analysis	Index	Impact
$\beta_{H}$	$U_{\beta_{H}}^{R_{0}}=\frac{\alpha_{0}\beta_{H}(d-z)}{\alpha_{0}\beta_{H}(d-z)+\beta_{Q}\phi }$	0.3589	Positive
Δ	$U_{\Delta }^{R_{0}}=1$	1.0000	Positive
σ	$U_{\sigma }^{R_{0}}=\frac{\xi }{(\xi +\sigma {)}^{2}}$	3.9233	Positive
$\alpha_{0}$	$U_{\alpha_{0}}^{R_{0}}=-\frac{\beta_{Q}\phi }{\alpha_{0}\beta_{H}(d-z)+\beta_{Q}\phi }$	−0.6411	Negative
$\beta_{Q}$	$U_{\beta_{Q}}^{R_{0}}=\frac{\beta_{Q}\phi }{\alpha_{0}\beta_{H}(d-z)+\beta_{Q}\phi }$	0.6411	Positive
ϕ	$U_{\phi }^{R_{0}}=\frac{\beta_{Q}\phi }{\alpha_{0}\beta_{H}(d-z)+\beta_{Q}\phi }$	0.6411	Positive
*d*	$U_{d}^{R_{0}}=-\frac{\beta_{Q}\phi d}{(d-z)\left[\alpha_{0}\beta_{H}(d-z)+\beta_{Q}\phi \right]}$	−0.7123	Negative
*z*	$U_{z}^{R_{0}}=\frac{\beta_{Q}\phi z}{(d-z)\left[\alpha_{0}\beta_{H}(d-z)+\beta_{Q}\phi \right]}$	0.0712	Positive
ρ	$U_{\rho }^{R_{0}}=-\frac{\rho }{\left(\xi +\rho +\delta \right)}$	−0.8739	Negative
δ	$U_{\delta }^{R_{0}}=-\frac{\delta }{\left(\xi +\rho +\delta \right)}$	−0.0952	Negative
ξ	$U_{\xi }^{R_{0}}=-\frac{\left[(\xi +\sigma )(\xi +\rho +\delta )+\xi (\xi +\rho +\delta )+\xi (\xi +\sigma )\right]}{\left(\xi +\rho +\delta )(\xi +\sigma \right)}$	−1.3076	Negative

The progression rate σ recorded the highest positive sensitivity index (3.9233), indicating that an increase in the rate at which exposed individuals become infectious significantly enhances disease spread. This implies that when infected individuals progress rapidly from the latent stage to the infectious stage, more people are able to shed *Salmonella Typhi* into the environment and transmit the infection to susceptible individuals. The recruitment rate Δ also positively affects *R*_0_ because continuous entry of susceptible individuals into the population increases the pool of individuals at risk of infection. Similarly, the environmental transmission parameters $\beta_{Q}$ and ϕ positively contribute to disease spread since typhoid fever is strongly associated with contaminated food and water sources; therefore, increased bacterial contamination in the environment enhances indirect transmission. The human-to-human transmission parameter $\beta_{H}$ also increases transmission, although moderately, because close contact with infected individuals can facilitate disease spread through poor hygiene practices. Conversely, the natural removal rate ξ has the strongest negative effect on *R*_0_, suggesting that increased natural mortality of individuals from the susceptible and infectious classes reduces the number of individuals available to sustain transmission. The treatment parameter ρ also negatively impacts transmission because effective treatment reduces the infectious period of infected individuals, thereby lowering bacterial shedding and disease spread. Furthermore, the disease-induced death rate δ contributes negatively to transmission since severely infected individuals who die are removed from the transmission chain. In general, the results emphasize that reducing environmental contamination, improving sanitation and hygiene, and ensuring early diagnosis and treatment are critical strategies for controlling typhoid fever transmission.

### Sensitivity assessment of endemic equilibrium

To better understand the key drivers of disease dynamics in system (2), we further performed a time-dependent sensitivity analysis at the endemic equilibrium. While classical approaches often focus on the basic reproduction number *R*_0_, analyzing sensitivities directly at the endemic equilibrium for all compartments provides a more detailed view of how changes in parameters affect the epidemic trajectory across different population states.

**Fig 4:**
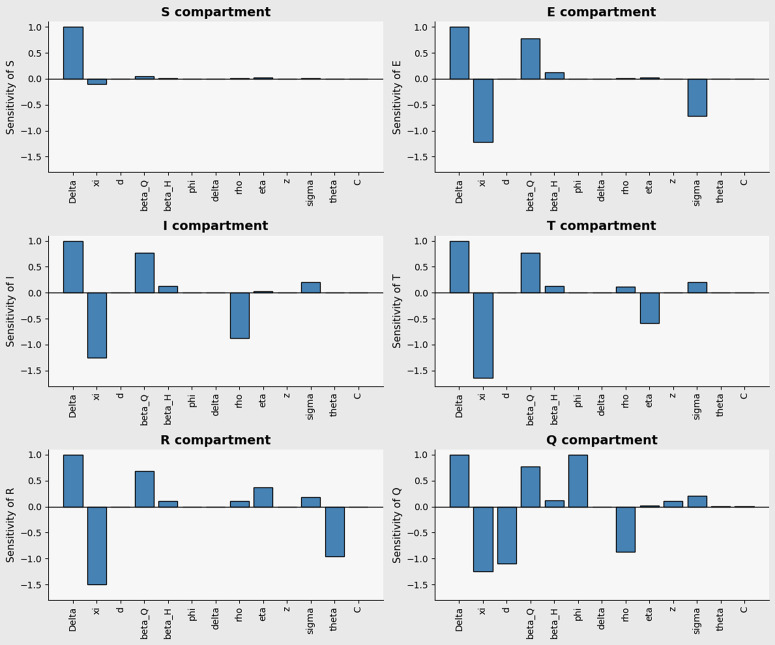
Normalized sensitivity indices of endemic equilibrium compartments.

Based on Equation (19) (see S1 File), for each compartment X∈{S,E,I,T,R,Q}, we computed the normalized sensitivity index as follows:


$$\begin{array}{c} U_{p}^{X}(t)=\frac{\partial X(t)}{\partial p}\cdot \frac{p}{X(t)}, \end{array}$$

which quantifies the relative change in the compartment *X* in response to a relative change in parameter *p* at the endemic state over time. Table 3 and Fig 4 depict this scenario.

**Table 3:** Normalized sensitivity indices of endemic equilibrium compartments using the updated parameter values. Positive (negative) values indicate parameters that increase (decrease) the corresponding endemic compartment.

Parameter	S^*^	E^*^	I^*^	T^*^	R^*^	Q^*^
Δ	1.0000	1.0000	1.0000	1.0000	1.0000	0.9941
ξ	−0.0955	−1.2205	−1.2518	−1.6458	−1.5026	−1.2445
*d*	0.0000	0.0000	0.0000	0.0000	0.0000	−1.0980
$\beta_{Q}$	0.0439	0.7742	0.7742	0.7742	0.6911	0.7697
$\beta_{H}$	0.0071	0.1252	0.1252	0.1252	0.1117	0.1244
ϕ	0.0000	0.0000	0.0000	0.0000	0.0000	0.9941
δ	0.0000	0.0000	0.0000	0.0000	0.0000	0.0000
ρ	0.0068	0.0068	−0.8797	0.1203	0.1073	−0.8746
η	0.0237	0.0237	0.0237	−0.5824	0.3728	0.0235
*z*	0.0000	0.0000	0.0000	0.0000	0.0000	0.1039
σ	0.0117	−0.7117	0.2062	0.2062	0.1841	0.2050
θ	0.0023	0.0023	0.0023	0.0023	−0.9645	0.0022
$\alpha_{0}$	0.0071	0.1252	0.1252	0.1252	0.1117	0.1244
$\alpha_{1}$	0.0000	0.0000	0.0000	0.0000	0.0000	0.0000
*C*	0.0000	0.0000	0.0000	0.0000	0.0000	0.0059

The sensitivity analysis of the endemic equilibrium compartments provides important biological insights into the long-term transmission dynamics of typhoid fever. The recruitment rate Δ exhibited a strong positive influence across all compartments, indicating that continuous entry of susceptible individuals into the population sustains disease persistence and maintains endemicity. The natural mortality rate ξ showed strong negative sensitivity across most compartments, particularly in the exposed $\left(E^{*}\right)$, infectious $\left(I^{*}\right)$, treated $\left(T^{*}\right)$, recovered $\left(R^{*}\right)$, and environmental pathogen $\left(Q^{*}\right)$ classes, implying that increasing the rate at which individuals leave the population reduces the number of infected individuals and consequently lowers environmental contamination. The environmental transmission parameter $\beta_{Q}$ and the direct transmission parameter $\beta_{H}$ positively influenced the exposed and infectious populations, demonstrating the dual role of contaminated water or food sources and direct human contact in sustaining typhoid transmission. The progression rate σ negatively affected the exposed class while positively influencing the infectious, treated, recovered, and environmental pathogen compartments, suggesting that faster progression from exposure to active infection increases bacterial shedding and disease burden. The treatment-related parameter ρ negatively impacted the infectious compartment but positively affected the treated and recovered populations, reflecting the epidemiological importance of prompt treatment in reducing active infection and enhancing recovery. Similarly, the recovery parameter η reduced the treated population while increasing the recovered class, indicating successful movement of individuals toward immunity. The immunity waning parameter θ strongly reduced the recovered compartment, showing that loss of immunity can return individuals to susceptibility and sustain endemic transmission. Furthermore, the environmental pathogen compartment $Q^{*}$ was highly sensitive to the bacterial shedding rate ϕ, environmental contamination parameter *z*, and bacterial decay parameter *d*. Specifically, increases in ϕ and *z* enhanced environmental contamination, whereas increasing *d* significantly reduced pathogen concentration in the environment. Biologically, these findings emphasize that typhoid fever persistence is strongly driven by environmental contamination, inadequate sanitation, continuous recruitment of susceptible individuals, and delayed treatment, highlighting the importance of clean water supply, hygiene practices, environmental sanitation, and rapid education and clinical intervention in controlling the disease.

### Optimal control analysis

We modify system (2) into an optimal control problem. We use the parameters *c*_1_ and *c*_2_ to represent educational awareness campaigns and treatment of water bodies respectively. Moreover, *c*_1_ and *c*_2_ are very effective if $c_{1}=c_{2}=1$ and ineffective when $c_{1}=c_{2}=0$. After incorporating the controls into system (2), we get the following:


$$\begin{array}{c} \left\{ \begin{array}{c} \frac{dS}{dt}=\Delta +\theta R-(1-c_{1})\Psi_{H}S-\xi S,\\ \frac{dE}{dt}=(1-c_{1})\Psi_{H}S-(\xi +\sigma )E,\\ \frac{dI}{dt}=\sigma E-(\xi +\delta +\rho )I,\\ \frac{dT}{dt}=\rho I-(\xi +\eta )T,\\ \frac{dR}{dt}=\eta T-(\xi +\theta )R,\\ \frac{dQ}{dt}=\lambda_{Q}+\phi I-c_{2}dQ. \end{array}\right. \end{array}$$ (6)

The associated control reproduction number for Eq (6) denoted by $R_{0}^{c}$ is obtained as


$$\begin{array}{c} R_{0}^{c}=\frac{\beta_{H}\sigma \;1mm\Delta \;1mm(1-c_{1})}{\xi \;1mm(\xi +\delta +\rho )\;1mm(\xi +\sigma )}+\frac{\beta_{Q}\;1mm\phi \sigma \;1mm\Delta \;1mm(1-c_{1})}{\xi \;1mm\alpha_{0}\;1mm(c_{2}\;1mmd-z)\;1mm(\xi +\delta +\rho )\;1mm(\xi +\sigma )}. \end{array}$$ (7)

The goal of this study is to minimize the number of exposed and infectious human populations (*E*, *I*), as well as the Salmonella Typhi bacteria population (*Q*) in the environment, while also minimizing the associated costs of implementing control measures *c*_1_ (educating susceptible individuals) and *c*_2_ (treating contaminated water). The objective function incorporates the social costs associated with these interventions, which are quadratic functions of the control variables: the cost of education is represented by $\frac{1}{2}f_{1}c_{1}^{2}$ and the cost of water treatment is represented by $\frac{1}{2}f_{2}c_{2}^{2}$.

The associated costs, *J*_1_, *J*_2_, and *J*_3_, represent the costs for minimizing the exposed human population *E*, the infected human population *I*, and the bacteria population in the environment *Q*, respectively. As shown in [30], the costs of minimizing *E*, *I*, and *Q* are linear, while the control costs $\frac{1}{2}f_{1}c_{1}^{2}$ and $\frac{1}{2}f_{2}c_{2}^{2}$ are quadratic. Therefore, the objective function is given by:


$$\begin{array}{c} J(c_{1},c_{2})=\int_{0}^{t_{f}}\left(J_{1}E+J_{2}I+J_{3}Q+\frac{1}{2}f_{1}c_{1}^{2}+\frac{1}{2}f_{2}c_{2}^{2}\right)dt, \end{array}$$

which is minimized over the time interval $\left[0,t_{f}\right]$, where $t_{f}$ represents the final intervention time.

To solve this optimal control problem and derive the necessary conditions, we apply Pontryagin’s Maximum Principle [30]. The Lagrangian is expressed as


$$\begin{array}{c} L=J_{1}E+J_{2}I+J_{3}Q+\frac{1}{2}f_{1}c_{1}^{2}+\frac{1}{2}f_{2}c_{2}^{2}. \end{array}$$

The Hamiltonian *H* for the control problem is defined as:


$$\begin{array}{c} H=J_{1}E+J_{2}I+J_{3}Q+\frac{1}{2}f_{1}c_{1}^{2}+\frac{1}{2}f_{2}c_{2}^{2}+\gamma_{S}\frac{dS}{dt}+\gamma_{E}\frac{dE}{dt}+\gamma_{I}\frac{dI}{dt}+\gamma_{T}\frac{dT}{dt}+\gamma_{R}\frac{dR}{dt}+\gamma_{Q}\frac{dQ}{dt}, \end{array}$$

where $\gamma_{S}$, $\gamma_{E}$, $\gamma_{I}$, $\gamma_{T}$, $\gamma_{R}$, and $\gamma_{Q}$ are the adjoint variables. The differential equations for these adjoint variables are derived by taking the partial derivatives of the Hamiltonian with respect to the state variables, as outlined in [31], yielding the following system of equations:


$$\begin{array}{c} \frac{d\gamma_{S}}{dt}=(\gamma_{S}-\gamma_{E})(1-c_{1})\left[\frac{\beta_{Q}Q}{Q+\alpha_{0}}+\frac{\beta_{H}I}{1+\alpha_{1}I}\right]+\gamma_{S}\xi , \end{array}$$

$$\begin{array}{c} \frac{d\gamma_{E}}{dt}=-J_{1}+(\gamma_{E}-\gamma_{I})\sigma +\gamma_{E}\xi , \end{array}$$

$$\begin{array}{c} \frac{d\gamma_{I}}{dt}=-J_{2}+(\gamma_{S}-\gamma_{E})(1-c_{1})\left[\frac{\beta_{H}S}{(1+\alpha_{1}I{)}^{2}}\right]+\gamma_{I}(\xi +\delta )+(\gamma_{I}-\gamma_{T})\rho -\gamma_{Q}\phi , \end{array}$$

$$\begin{array}{c} \frac{d\gamma_{T}}{dt}=(\gamma_{T}-\gamma_{R})\eta +\gamma_{T}\xi , \end{array}$$

$$\begin{array}{c} \frac{d\gamma_{R}}{dt}=(\gamma_{R}-\gamma_{S})\theta +\gamma_{R}\xi , \end{array}$$

$$\begin{array}{c} \frac{d\gamma_{Q}}{dt}=-J_{3}+(\gamma_{S}-\gamma_{E})(1-c_{1})\left[\frac{\beta_{Q}S\alpha_{0}}{(Q+\alpha_{0}{)}^{2}}\right]+\gamma_{Q}dc_{2}+\gamma_{Q}z\left[\frac{2Q}{C}-1\right]. \end{array}$$

**Theorem 1.** *If there exists an optimal control pair* $c_{1}^{*}$ *and* $c_{2}^{*}$, and corresponding state variables S, E, I, T, R, and Q that minimize the objective function $J(c_{1},c_{2})$ *over the region* Ω*, then there exist adjoint variables* $\gamma_{S}$*,* $\gamma_{E}$*,* $\gamma_{I}$*,* $\gamma_{T}$*,* $\gamma_{R}$*, and* $\gamma_{Q}$ *that satisfy:*


$$\begin{array}{c} \frac{d\gamma_{i}}{dt}=-\frac{\partial H}{\partial i},\;i\in \{S,E,I,T,R,Q\}. \end{array}$$

*Thus, the optimal control pair* $c_{1}^{*}$, $c_{2}^{*}$ *can be determined as follows:*


$$\begin{array}{c} c_{1}^{*}=\min\left(1,\max(0,{\hat{c}}_{1})\right), \end{array}$$

$$\begin{array}{c} c_{2}^{*}=\min\left(1,\max(0,{\hat{c}}_{2})\right). \end{array}$$

**Proof 1** *The adjoint variables and transversality conditions are standard results from Pontryagin’s Maximum Principle* [31]. *By applying the optimality conditions:*


$$\begin{array}{c} \frac{\partial H}{\partial c_{1}}=0,\;\frac{\partial H}{\partial c_{2}}=0, \end{array}$$

*we obtain the following equations:*


$$\begin{array}{c} f_{1}c_{1}+(\gamma_{S}-\gamma_{E})\left[\frac{\beta_{Q}SQ}{Q+\alpha_{0}}+\frac{\beta_{H}SI}{1+\alpha_{1}I}\right]=0, \end{array}$$

$$\begin{array}{c} f_{2}c_{2}-\gamma_{Q}dQ=0. \end{array}$$

*Solving for c*_*1*_ *and c*_*2*_*, we get:*


$$\begin{array}{c} {\hat{c}}_{1}=\frac{\left(\gamma_{E}-\gamma_{S}\right)}{f_{1}}\left[\frac{\beta_{Q}SQ}{Q+\alpha_{0}}+\frac{\beta_{H}SI}{1+\alpha_{1}I}\right], \end{array}$$

$$\begin{array}{c} {\hat{c}}_{2}=\frac{\gamma_{Q}dQ}{f_{2}}. \end{array}$$

*Given that the control parameters are bounded by 0 and 1, the optimal control values* $c_{1}^{*}$ *and* $c_{2}^{*}$ *are computed as:*


$$\begin{array}{c} c_{1}^{*}=\left\{ \begin{array}{cc} {\hat{c}}_{1} & \text{if }0<{\hat{c}}_{1}<1,\\ 0 & \text{if }{\hat{c}}_{1}\leq 0,\\ 1 & \text{if }{\hat{c}}_{1}\geq 1. \end{array}\right. \end{array}$$

$$\begin{array}{c} c_{2}^{*}=\left\{ \begin{array}{cc} {\hat{c}}_{2} & \text{if }0<{\hat{c}}_{2}<1,\\ 0 & \text{if }{\hat{c}}_{2}\leq 0,\\ 1 & \text{if }{\hat{c}}_{2}\geq 1. \end{array}\right. \end{array}$$

*Thus, the optimal control parameters* $c_{1}^{*}$ *and* $c_{2}^{*}$ *minimize the objective function* $J(c_{1},c_{2})$*, and their corresponding values are computed numerically using Python.*

## Results

### Optimal control analysis results

Numerical simulations of system (6) were performed using R and Octave, with parameter values provided in Table 1. The optimal control problem was solved using the fourth-order Runge–Kutta method. Initial conditions were set as: *S*(0) = 870492, *E*(0) = 20000, *I*(0) = 1208, *T*(0) = 900, *R*(0) = 300, and *Q*(0) = 500. The state and control coefficients used were *J*_1_ = 1, *J*_2_ = 5, *J*_3_ = 10, *f*_1_ = 2, and *f*_2_ = 10.

To assess the impact of each control on the eradication of Typhoid fever in the population and environment, we implemented the following strategies:

### Control with educational campaigns only

**Fig 5:**
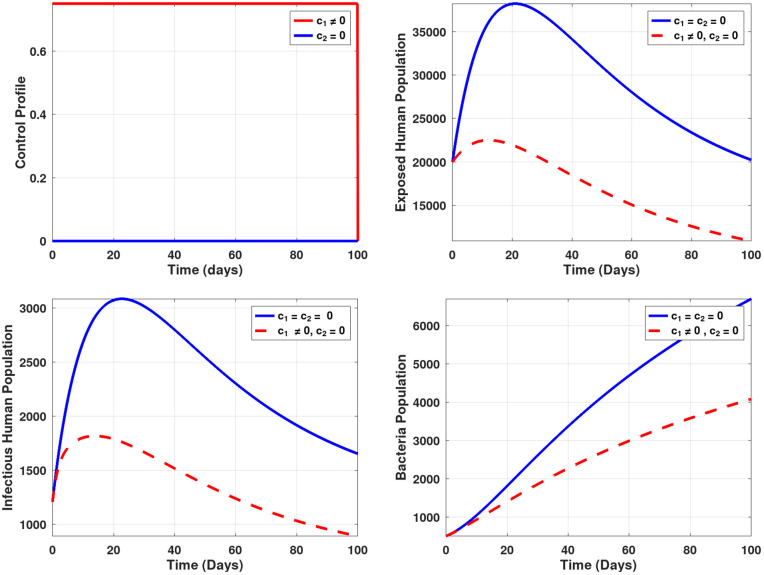
Effect of educational awareness campaigns only. Control graph shows intensifying awareness campaign for 100 days effectively reduces exposed, infected and bacterial population.

In this strategy, we focus solely on educational campaigns ($c_{1}\neq 0$ and *c*_2_ = 0) as the control mechanism to minimize the objective function (*see* Fig 5).

The goal is to reduce Typhoid fever transmission by promoting awareness about safe food handling, proper hygiene practices (such as frequent handwashing), disinfection of commonly used surfaces, responsible coughing and sneezing, avoiding the sharing of personal items, vaccination, and early treatment. The time frame for this study is set at 100 days. The control profile suggests that continuous education of all individuals is necessary to significantly reduce transmission of the disease. This approach effectively lowers the number of individuals exposed to Typhoid fever. Since infected individuals are key drivers of disease transmission and environmental contamination, educating the population on causes and prevention of Typhoid fever helps mitigate spread of the bacteria. Moreover, educating individuals has a substantial impact on reducing environmental contamination. Educated individuals are more likely to adopt positive behavior changes, leading to a decrease in bacterial levels in the environment.

### Controlling Typhoid with treatment of water bodies only

**Fig 6:**
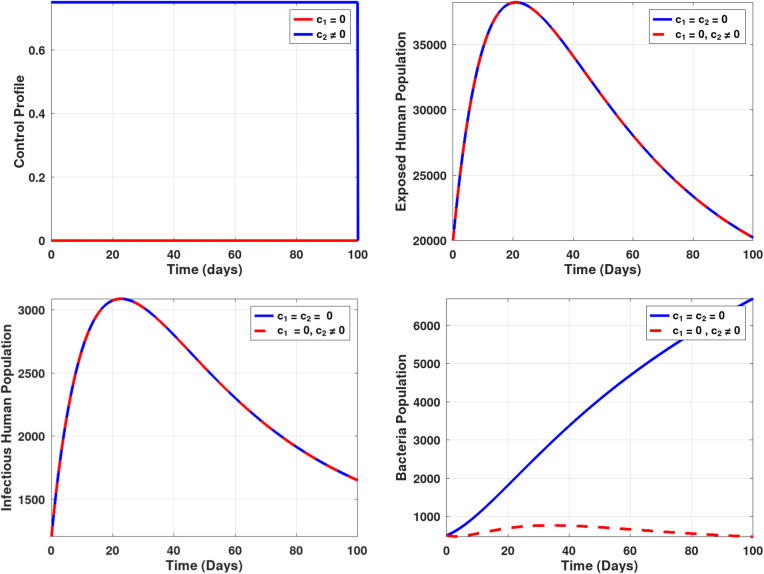
Effect of water treatment only. Control graph shows treating water bodies for 100 days effectively reduces bacterial population but insignificant on exposed and infected population.

The strategy in Fig 6 evaluates the effectiveness of treating water bodies as the sole control measure, with *c*_1_ = 0 and $c_{2}\neq 0$.

It was observed that treating water bodies alone has minimal impact on the number of exposed and infected individuals. This is because human-to-human transmission is the primary mode of Typhoid fever, and without addressing this, water treatment alone cannot significantly reduce disease spread. In a fully infected population, where individuals do not change their behavior, water treatment alone shows limited results. Although untreated water bodies lead to an increase in the bacterial population, treatment of these water bodies can reduce bacteria growth by decreasing the rate of bacterial proliferation. When applied consistently, treating contaminated water sources daily helps minimize the bacterial load in the environment, making it an effective intervention for controlling environmental contamination.

### Controlling Typhoid with both educational awareness campaign and treatment of water bodies

**Fig 7:**
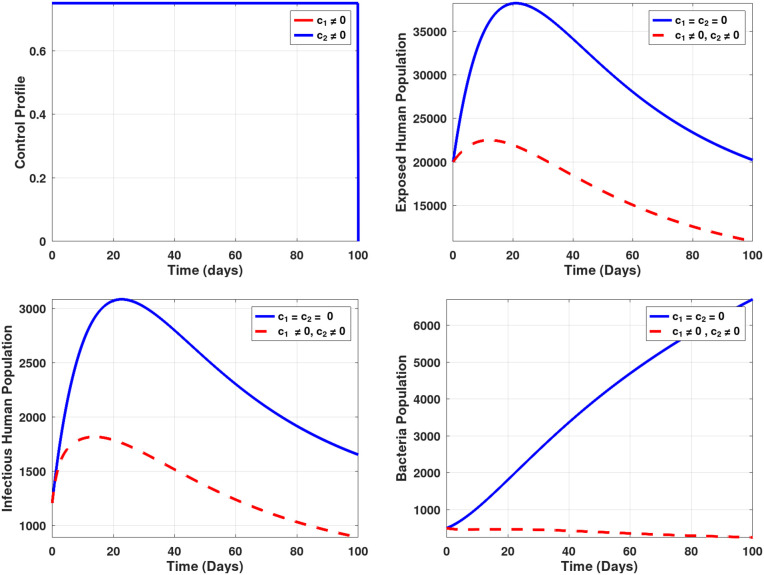
Effect of combined controls. Daily education for all exposed and infected individuals, together with continuous treatment of water bodies over 100 days, is essential for eradicating bacteria population.

The strategy in Fig 7 evaluates the combined effect of educational campaigns and water body treatment as an intervention for reducing Typhoid fever transmission.

This strategy shows that with both controls in place, exposed individuals gain awareness of the causes, transmission modes, and effects of Salmonella Typhi, leading to better hygiene practices (e.g., handwashing, avoiding contact with infected individuals) and improved sanitation habits. This reduces exposure to the disease within the population. Infected individuals also benefit from educational campaigns, which inform them about behaviors that contribute to disease transmission and the importance of seeking treatment. This decreases the number of infected individuals and lowers the risk of reinfection through contaminated water sources. Simultaneously treating water bodies also lowers bacterial contamination in the environment, which further reduces exposure to the bacteria. Without proper education and safe drinking water, Typhoid fever becomes persistent in the population. However, when both controls are applied, bacterial concentrations in the environment decrease, and individuals gain access to safe drinking water and the implementation of educational campaigns further reduce disease spread by promoting proper sanitation.

## Conclusion

This study presents an integrated mathematical framework for modeling Typhoid fever transmission, incorporating both human-to-human dynamics and environmental contamination. Our findings highlight the significant role of environmental transmission, particularly the contact rate between humans and bacteria in contaminated water, in driving Typhoid fever transmission. Through sensitivity analysis, we confirmed that interventions targeting both human behavior, using educational awareness campaigns, and environmental factors, through water treatment, are crucial for effective disease control. While educational campaigns alone significantly reduced both human-to-human transmission and environmental contamination, water treatment alone proved effective in reducing bacterial load in the environment. Combining these strategies was the most successful in reducing disease transmission. These results suggest that a multi-faceted approach, addressing both environmental and behavioral factors, is essential for Typhoid fever control in endemic regions. This study is limited by its deterministic framework, which assumes continuous interactions and population dynamics. Future work could employ a stochastic modeling approach, treating the environmental bacterial population as discrete and interactions as non-continuous, potentially providing more realistic dynamics and improved predictive power. In addition, the model assumes homogeneous mixing of the population, implying that all individuals have equal contact with contaminated water sources and infected individuals, which may not accurately reflect real-world contact patterns. The model also does not incorporate spatial heterogeneity, such as geographic differences in sanitation infrastructure, population density, and access to clean water, which can significantly influence disease transmission dynamics. Furthermore, the model adopts simplified representations of human behavior and intervention uptake, thereby limiting behavioral realism, particularly with respect to compliance with public health education and environmental sanitation measures.

## Supporting information

S1 File Supporting information. (PDF)

## References

[1] Akullian A, Ng’eno E, Matheson AI, Cosmas L, Macharia D, Fields B, et al. Environmental Transmission of Typhoid Fever in an Urban Slum. PLoS Negl Trop Dis. 2015;9(12):e0004212. doi: 10.1371/journal.pntd.0004212 26633656 PMC4669139

[2] Paul UK, Bandyopadhyay A. Typhoid fever: a review. Int J Adv Med. 2017;4(2):300.

[3] Pitzer VE, Meiring J, Martineau FP, Watson CH, Kang G, Basnyat B, et al. The Invisible Burden: Diagnosing and Combatting Typhoid Fever in Asia and Africa. Clin Infect Dis. 2019;69(Suppl 5):S395–401. doi: 10.1093/cid/ciz611 31612938 PMC6792124

[4] Andrews JR, Vaidya K, Saha S, Yousafzai MT, Hemlock C, Longley A, et al. Healthcare Utilization Patterns for Acute Febrile Illness in Bangladesh, Nepal, and Pakistan: Results from the Surveillance for Enteric Fever in Asia Project. Clin Infect Dis. 2020;71(Suppl 3):S248–56. doi: 10.1093/cid/ciaa1321 33258937 PMC7705868

[5] Asadi F, Trinugroho JP, Hidayat AA, Rahutomo R, Pardamean B. Data mining for epidemiology: The correlation of typhoid fever occurrence and environmental factors. Procedia Computer Science. 2023;216:284–92. doi: 10.1016/j.procs.2022.12.138

[6] Nthiiri J. Mathematical modelling of typhoid fever disease incorporating protection against infection. 2016.

[7] Khanam F, Ross AG, McMillan NAJ, Qadri F. Toward Typhoid Fever Elimination. Int J Infect Dis. 2022;119:41–3. doi: 10.1016/j.ijid.2022.03.036 35338009

[8] Edward S, et al. A deterministic mathematical model for direct and indirect transmission dynamics of typhoid fever. Open Access Library Journal. 2017;4(05):1.

[9] Peter O, Afolabi O, Oguntolu F, Ishola C, Victor A. Solution of a deterministic mathematical model of typhoid fever by variational iteration method. Science World Journal. 2018;13(2):64–8.

[10] Musa SS, Zhao S, Hussaini N, Usaini S, He D. Dynamics analysis of typhoid fever with public health education programs and final epidemic size relation. Results in Applied Mathematics. 2021;10:100153. doi: 10.1016/j.rinam.2021.100153

[11] Khan MA, Parvez M, Islam S, Khan I, Shafie S, Gul T. Mathematical analysis of typhoid model with saturated incidence rate. asb. 2015;7:65–78. doi: 10.12988/asb.2015.41059

[12] Pitzer VE, Feasey NA, Msefula C, Mallewa J, Kennedy N, Dube Q, et al. Mathematical Modeling to Assess the Drivers of the Recent Emergence of Typhoid Fever in Blantyre, Malawi. Clin Infect Dis. 2015;61 Suppl 4(Suppl 4):S251-8. doi: 10.1093/cid/civ710 26449939 PMC4596932

[13] Edward S, Nyerere N. Modelling typhoid fever with education, vaccination and treatment. Eng Math. 2016;1(1):44–52.

[14] Nyerere N, Mpeshe SC, Edward S. Modeling the impact of screening and treatment on the dynamics of typhoid fever. World Journal of Modelling and Simulation. 2018;14(4):298–306.

[15] Peter OJ, Ibrahim MO, Edogbanya HO, Oguntolu FA, Oshinubi K, Ibrahim AA, et al. Direct and indirect transmission of typhoid fever model with optimal control. Results in Physics. 2021;27:104463. doi: 10.1016/j.rinp.2021.104463 PMC8323584 34345579

[16] Burrows H, Antillón M, Gauld JS, Kim J-H, Mogasale V, Ryckman T, et al. Comparison of model predictions of typhoid conjugate vaccine public health impact and cost-effectiveness. Vaccine. 2023;41(4):965–75. doi: 10.1016/j.vaccine.2022.12.032 36586741 PMC9880559

[17] Irena TK, Gakkhar S. Modeling the role of vaccination, environmental sanitation, and saturated treatment on the spread of typhoid fever. J Biol Syst. 2022;30(02):459–95. doi: 10.1142/s0218339022500164

[18] Kailan Suhuyini A, Seidu B. A mathematical model on the transmission dynamics of typhoid fever with treatment and booster vaccination. Front Appl Math Stat. 2023;9. doi: 10.3389/fams.2023.1151270

[19] Tilahun GT, Makinde OD, Malonza D. Modelling and Optimal Control of Typhoid Fever Disease with Cost-Effective Strategies. Comput Math Methods Med. 2017;2017:2324518. doi: 10.1155/2017/2324518 29081828 PMC5610837

[20] Abboubakar H, Racke R. Mathematical modeling, forecasting, and optimal control of typhoid fever transmission dynamics. Chaos, Solitons & Fractals. 2021;149:111074. doi: 10.1016/j.chaos.2021.111074

[21] Khan IU, Mustafa S, Shokri A, Li S, Akgül A, Bariq A. The stability analysis of a nonlinear mathematical model for typhoid fever disease. Sci Rep. 2023;13(1):15284. doi: 10.1038/s41598-023-42244-5 37714901 PMC10504385

[22] Dayan F, Ahmed N, Ali AH, Rafiq M, Raza A. Numerical investigation of a typhoid disease model in fuzzy environment. Sci Rep. 2023;13(1):21993. doi: 10.1038/s41598-023-48405-w 38081842 PMC10713662

[23] Lawal FO, Yusuf TT, Abidemi A, Olotu O. A non-linear mathematical model for typhoid fever transmission dynamics with medically hygienic compartment. Model Earth Syst Environ. 2024;10(5):6213–32. doi: 10.1007/s40808-024-02111-2

[24] Sharma L, Laxman, Kumar R. Transmission dynamics of typhoid fever with nonlinear incidence rate and saturated treatment. Int J Dynam Control. 2025;14(1). doi: 10.1007/s40435-025-01953-7

[25] Nana-Kyere S, Asamoah KK, De-Graft Ankamah J, Okyere E, Seidu B, Kwarteng D. Mathematical Modeling and Cost-Effectiveness Analysis of an SeEeIeRe Typhoid Fever Model. Journal of Mathematics. 2025;2025(1):1212057.

[26] Boakye Okyere P, Twumasi-Ankrah S, Newton S, Nkansah Darko S, Owusu Ansah M, Darko E, et al. Risk Factors for Typhoid Fever: Systematic Review. JMIR Public Health Surveill. 2025;11:e67544. doi: 10.2196/67544 40875987 PMC12426575

[27] Singer S, Nelder J. Nelder-Mead algorithm. Scholarpedia. 2009;4(7):2928. doi: 10.4249/scholarpedia.2928

[28] Zhou N, Ong A, Fagnant-Sperati C, Harrison J, Kossik A, Beck N, et al. Evaluation of Sampling and Concentration Methods for Salmonella enterica Serovar Typhi Detection from Wastewater. Am J Trop Med Hyg. 2023;108(3):482–91. doi: 10.4269/ajtmh.22-0427 36746655 PMC9978546

[29] Irunde JI, Ndendya JZ, Mwasunda JA, Robert PK. Modeling the impact of screening and treatment on typhoid fever dynamics in unprotected population. Results in Physics. 2023;54:107120. doi: 10.1016/j.rinp.2023.107120

[30] Lewis AD. The maximum principle of Pontryagin in control and in optimal control. 2006.

[31] Opoku NK-DO, Borkor RN, Adu AF, Nyarko HN, Doughan A, Appiah EM, et al. Modelling the Transmission Dynamics of Meningitis among High and Low-Risk People in Ghana with Cost-Effectiveness Analysis. Abstract and Applied Analysis. 2022;2022:1–24. doi: 10.1155/2022/9084283

