# Supplementary material for: Optimal control of Typhoid fever transmission under environmental and public health interventions
Source: PLoS One. 2026 Jun 24;21(6):e0351747. doi: 10.1371/journal.pone.0351747 (PMC13293465; doi:10.1371/journal.pone.0351747)
Supplement: S1 File — (PDF) [file pone.0351747.s001.pdf]

Supplementary Material

1 Analysis of the Model

$$
\begin{cases}
\dfrac{dS}{dt} &= \Delta + \theta R - \Psi_H S - \xi S, \\[2ex]
\dfrac{dE}{dt} &= \Psi_H S - (\xi + \sigma)E, \\[2ex]
\dfrac{dI}{dt} &= \sigma E - (\xi + \delta + \rho)I, \\[2ex]
\dfrac{dT}{dt} &= \rho I - (\xi + \eta)T, \\[2ex]
\dfrac{dR}{dt} &= \eta T - (\xi + \theta)R, \\[2ex]
\dfrac{dQ}{dt} &= \lambda_Q + \phi I - dQ.
\end{cases} \tag{1}
$$

1.1 Positivity of solutions

Theorem 1. Given the initial conditions $S(0) > 0$, $E(0) \geq 0$, $I(0) \geq 0$, $T(0) \geq 0$, $R(0) \geq 0$, and $Q(0) \geq 0$, then the solutions of $S(t)$, $E(t)$, $I(t)$, $T(t)$, $R(t)$, and $Q(t)$ of system (1) are positive for all $t \geq 0$.

Proof. Assume that $\hat{t}$ = $\sup\{t > 0 : S(0) > 0,\ E(0) \geq 0,\ I(0) \geq 0,\ T(0) \geq 0,\ R(0) \geq 0, \text{ and } Q \geq 0\} \in [0, t]$, this implies that $S(0) > 0$ and $E(0) \geq 0$, $I(0) \geq 0$, $T(0) \geq 0$, $R(0) \geq 0$, and $Q \geq 0$, $\forall t \in [0, t]$, and $\hat{t} \geq 0$. Considering the first equation system (1), we have:

$$\frac{dS}{dt} = \Delta - \xi S - \Psi_H + \theta R.$$

Since Δ and θR are non-negative then:

$$\frac{dS}{dt} \geq -\xi S - \Psi_H.$$

By separation of variables and integration of both sides:

$$S(\hat{t}) \geq S(0) \exp\left(-\xi\hat{t} - \int_0^{\hat{t}} \left[\frac{\beta_Q\, Q}{Q + \alpha_0} + \frac{\beta_H\, I}{1 + \alpha_1 I}\right] dt\right) > 0.$$

Since $S(0) > 0$, we can see clearly that $S(\hat{t})$ will always be positive even for non-constant force of infection Ψ_H. Similarly, the same procedure can be used to show that $E(\hat{t}) \geq 0, I(\hat{t}) \geq 0, T(\hat{t}) > 0, R(\hat{t}) > 0$, and $Q(\hat{t}) > 0$. Therefore, $S(t) > 0$, $E(t)\geq 0$, $I(t)\geq 0$, $T(t)\geq 0$, $R(t)\geq 0$, and $Q(t) \geq 0$ for all $t \geq 0$, given the initial conditions. As a result, the solution of system 1 is positive. □

1.2 Boundedness of Solution

Theorem 2. The biologically feasible region, Ω, for system (1) is given by the following invariant region:

$$\Omega = \{(S(t), E(t), I(t), T(t), R(t), Q(t)) \in \mathbb{R}^6_+ \mid S + E + I + T + R \leq \frac{\Delta}{\xi},\ 0 \leq Q \leq \hat{Q}\}, \tag{2}$$

where $\hat{Q}$ is the maximum number of the bacteria in the environment, and $d - z > 0$. The basic properties of local existence, uniqueness, and continuity of the solutions are valid for system (1), with non-negative initial conditions $S(0) > 0$, $E(0) \geq 0$, $I(0) \geq 0$, $T(0) \geq 0$, $R(0) \geq 0$, and $Q(0) \geq 0$. Therefore, the set Ω is bounded, positively invariant, and attracting with respect to system (1) for all $t > 0$.

Proof. To get dynamics of total human population $\frac{dN}{dt}$, we sum up the system (1) for humans to obtain:

$$\frac{dN}{dt} = \Delta - \xi(S + E + I + T + R) - \delta I.$$

For $N(t) = S(t) + E(t) + I(t) + T(t) + R(t)$, we obtain;

$$\begin{aligned}\frac{dN}{dt} &= \Delta - \xi N - \delta I,\\ \frac{dN}{dt} &\leq \Delta - \xi N. \end{aligned} \tag{3}$$

The solution of equation (3) is given by:

$$N(t) \leq \frac{\Delta}{\xi} + Ae^{-\xi t}, \tag{4}$$

where A is a constant.Considering the initial conditions in equation (4) at $t = 0$, let $N(0)$ be the initial total human population which is the sum of all the initial conditions of system (1) for the human population. Therefore,

$$\begin{aligned} N(0) &\leq \frac{\Delta}{\xi} + A,\\ A &\geq N(0) - \frac{\Delta}{\xi}. \end{aligned}$$

Hence,

$$N(t) \leq \frac{\Delta}{\xi} + \left(N(0) - \frac{\Delta}{\xi}\right) e^{-\xi t}. \tag{5}$$

As $N(t) \to \frac{\Delta}{\xi}$, every solution $N(t)$ in equation (3) satisfies the following condition:

$$0 \leq N(t) \leq N(0)e^{-\xi t} + \left(1 - e^{-\xi t}\right) \frac{\Delta}{\xi}.$$

Therefore, the total human population at time, t, is bounded above by $\frac{\Delta}{\xi}$ if $N(0) \leq \frac{\Delta}{\xi}$. Hence, for any solution of system (1), it either remains in or approaches Ω asymptotically. Therefore,

$$S(t) \leq \frac{\Delta}{\xi}, \quad E(t) \leq \frac{\Delta}{\xi}, \quad I(t) \leq \frac{\Delta}{\xi}, \quad T(t) \leq \frac{\Delta}{\xi}, \quad R(t) \leq \frac{\Delta}{\xi}.$$

Next, we consider the boundedness of $Q(t)$, by computing the equilibrium solution of Q:

$$0 = \lambda_Q + \phi I - dQ. \tag{6}$$

Since $\lambda_Q = zQ\left(1 - \frac{Q}{C}\right)$, then equation (6) becomes:

$$zQ^2 + C(d - z)Q - C\phi I = 0, \tag{7}$$

where $d - z > 0$. The state $\hat{I}$ is an upper bound for the number of infected individuals. Therefore,

$$Q_{1,2} = \frac{-(Cd - Cz) \pm \sqrt{(Cd - Cz)^2 + 4Cz\phi\hat{I}}}{2z},$$

where the negative root always give the smallest value, and the positive root gives the larger value. From the boundedness, $\hat{I} \leq \frac{\Delta}{\xi}$, we can conclude that Q is always non-negative and bounded by:

$$\hat{Q} = \frac{-(Cd - Cz) + \sqrt{(Cd - Cz)^2 + 4Cz\phi\hat{I}}}{2z} < \infty,$$

since the derivative of the state equation $\frac{dQ}{dt}$ is always non-negative for $Q \leq \hat{Q}$ and negative for all value of $Q > \hat{Q}$. Therefore, Ω is positively invariant and attractive with respect to the model. □

1.3 Model Equilibrium points

1.3.1 Disease-Free Equilibrium (DFE) and Basic Reproduction Number

The disease free equilibrium (DFE) state is obtained when the population remains stable in the absence of Typhoid fever disease and Salmonella Typhi bacteria in the environment. If we denote the DFE by E_0 together with the conditions $E = I = T = R = Q = 0$, then we get

$$E_0 = (S^0, E^0, I^0, T^0, R^0, Q^0) = \left(\frac{\Delta}{\xi}, 0, 0, 0, 0, 0\right). \tag{8}$$

Epidemiological models usually have a threshold parameter known as the basic reproduction number, R_0 [1]. In the context of Typhoid fever, R_0 is the expected number of secondary cases that occur from one infected individual or Salmonella Typhi bacteria introduced into an otherwise completely susceptible population or environment [1]. To derive R_0, we use the standard next-generation matrix theory applied in [1] to get

$$F = \begin{pmatrix} 0 & \frac{\Delta\beta_H}{\xi} & \frac{\Delta\beta_Q}{\xi\alpha_0} \\ 0 & 0 & 0 \\ 0 & 0 & 0 \end{pmatrix}, \tag{9}$$

and

$$V = \begin{pmatrix} (\xi+\sigma) & 0 & 0 \\ -\sigma & (\xi+\delta+\rho) & 0 \\ 0 & -\phi & (d-z) \end{pmatrix}. \tag{10}$$

The basic reproduction number of system (1) can be considered as the spectral radius (the maximum eigenvalue) of the next-generational matrix FV^{-1} [1]. Therefore;

$$R_0 = \frac{\beta_H \sigma\ \Delta}{\xi\ (\xi+\delta+\rho)\ (\xi+\sigma)} + \frac{\beta_Q\ \phi\sigma\ \Delta}{\xi\ \alpha_0\ (d-z)\ (\xi+\delta+\rho)\ (\xi+\sigma)}. \tag{11}$$

The basic reproduction number is seen to be the aggregation of infected human population and the density of Salmonella Typhi bacteria in the environment.

1.3.2 Local Stability of the Disease-Free Equilibrium

Theorem 3. If $R_0 < 1$, the DFE state is locally asymptotically stable in the region defined by $\Omega = \{(S, E, I, T, R, Q) \in \mathbb{R}^6_+ \mid S + E + I + T + R \leq \frac{\Delta}{\xi},\ 0 \leq Q \leq \hat{Q}\}$ and unstable if $R_0 > 1$.

Proof. We linearize system (1) around the DFE to obtain:

$$J_{E_0} = \begin{pmatrix} -\xi & 0 & -\frac{\Delta\ \beta_H}{\xi} & 0 & \theta & -\frac{\Delta\ \beta_Q}{\xi\ \alpha_0} \\ 0 & -(\xi+\sigma) & \frac{\Delta\ \beta_H}{\xi} & 0 & 0 & \frac{\Delta\ \beta_Q}{\xi\ \alpha_0} \\ 0 & \sigma & -(\xi+\delta+\rho) & 0 & 0 & 0 \\ 0 & 0 & \rho & -(\xi+\eta) & 0 & 0 \\ 0 & 0 & 0 & \eta & -(\xi+\theta) & 0 \\ 0 & 0 & \phi & 0 & 0 & -(d-z) \end{pmatrix}. \tag{12}$$

Per the basic properties of matrix algebra as shown in [2], we observed that the eigenvalues $\lambda_1 = -\xi$, $\lambda_5 = -(\xi+\theta)$ and $\lambda_4 = -(\xi+\eta)$ of equation (12) have negative real parts. The reduced matrix of J_{E_0} becomes

$$J^*_{E_0} = \begin{pmatrix} -(\xi+\sigma) & \dfrac{\Delta\beta_H}{\xi} & \dfrac{\Delta\beta_Q}{\xi\alpha_0} \\ \sigma & -(\xi+\delta+\rho) & 0 \\ 0 & \phi & -(d-z) \end{pmatrix}. \tag{13}$$

The matrix $J^*_{E_0}$ can be written as $J^*_{E_0} = F - V$, where F and V are given by the matrices of equations (9) and (10). Given that $(\xi+\sigma)$, $(\xi+\delta+\rho)$ and $(d-z)$ are positive and $R_0 \le 1$ is sufficient for the matrix $J^*_{E_0}$ to have eigenvalues with real parts less or equal to zero. Hence, the DFE is locally asymptotically stable.

□

1.3.3 Global Stability of Disease-Free Equilibrium

Theorem 4. The DFE, E_0, is globally asymptotically stable in the region defined by $\Omega = \{(S,E,I,T,R,Q) \in \mathbb{R}^6_+ \mid S+E+I+T+R \le \dfrac{\Delta}{\xi},\ 0 \le Q \le \hat{Q}\}$ if $R_0 < 1$ and unstable if $R_0 > 1$.

Proof. Applying the method in [2], we define a Lyapunov function

$$K = \theta_1 E + \theta_2 I + \theta_3 Q, \tag{14}$$

where $\theta_1, \theta_2, \theta_3 > 0$ are constants to be determine. Differentiating equation (14) gives

$$\frac{dK}{dt} = \theta_1 \frac{dE}{dt} + \theta_2 \frac{dI}{dt} + \theta_3 \frac{dQ}{dt}. \tag{15}$$

Substitute the value of $\dfrac{dE}{dt}$, $\dfrac{dI}{dt}$ and $\dfrac{dQ}{dt}$ into equation (15) gives

$$\begin{aligned}\frac{dK}{dt} &= \theta_1(\Psi_H - E(\xi+\sigma)) + \theta_2(\sigma E - I(\xi+\rho+\delta)) + \theta_3(\lambda_Q + \phi I - dQ),\\ &= \theta_1\left(\frac{\beta_H S I}{1+\alpha_1 I}\right) - \theta_2(\xi+\rho+\delta)I + \theta_3\phi I - \theta_1(\xi+\sigma)E + \theta_2\sigma E + \theta_1\left(\frac{\beta_Q S Q}{Q+\alpha_0}\right) + \theta_3(\lambda_Q - dQ).\end{aligned} \tag{16}$$

By grouping terms for E , Q and I in equation (16), we get

$$\frac{dK}{dt} = \left(\frac{\beta_H S\theta_1}{1+\alpha_1 I} - \theta_2(\xi+\rho+\delta) + \theta_3\phi\right) I + (\theta_2\sigma - \theta_1(\xi+\sigma))\,E + \left(\frac{\beta_Q S\theta_1}{Q+\alpha_0} + \theta_3\left(z - \frac{zQ}{C} - d\right)\right) Q.$$

$$\frac{dK}{dt} \le \left(\frac{\Delta\,\beta_H}{\xi}\theta_1 - \theta_2(\xi+\rho+\delta) + \theta_3\phi\right) I + (\theta_2\sigma - \theta_1(\xi+\sigma))\,E + \left(\frac{\Delta\,\beta_Q}{\xi\,\alpha_0}\theta_1 + \theta_3\,(z-d)\right) Q. \tag{17}$$

Equating the coefficients of Q and E in equation (17) to zero which correspond to the DFE and applying the multiplication property of equality which states that if $u = v$ and $w = x$, then $uw = vx$, we obtain

$$\frac{\Delta\,\beta_Q}{\xi\,\alpha_0}\theta_1 = \theta_3\,(d-z)\,,$$

and

$$\theta_2\sigma = \theta_1(\xi+\sigma). \tag{18}$$

This implies that

$$\frac{\Delta\,\beta_Q}{\xi\,\alpha_0}\,\sigma\,\theta_2 = \theta_3\,(d-z)\,(\xi+\sigma).$$

Choosing $\theta_2 = (d-z)\,(\xi+\sigma)$, $\theta_3 = \dfrac{\Delta\sigma\beta_Q}{\xi\ \alpha_0}$ and substituting them into equation (18) gives

$$\theta_1 = \sigma(d-z).$$

Further substituting the values of θ_1, θ_2, and θ_3 in equation (17) we get

$$\frac{dK}{dt} \leq \left(\frac{\Delta\ \beta_H\ \sigma}{\xi}(d-z) - (d-z)\,(\xi+\sigma)(\xi+\rho+\delta) + \frac{\Delta\ \beta_Q\ \phi\ \sigma}{\xi\ \alpha_0}\right) I.$$

After some algebraic calculations, we obtain

$$\frac{dK}{dt} \leq (R_0 - 1)\,(d-z)\,(\xi+\sigma)(\xi+\rho+\delta)I.$$

Therefore, $\dfrac{dK}{dt} = 0$ if $I = 0$ and $\dfrac{dK}{dt} < 0$ if $R_0 < 1$. As result, we set the largest compact invariant in $\{(S,E,I,T,R,Q) \in \Omega : \dfrac{dK}{dt}(E,I,Q) = 0\}$ is the singleton set $\{E_0\}$. From La Salle's invariance principle ([3]), it can be concluded that E_0 is globally asymptotically stable in Ω if $R_0 < 1$. $\square$

1.4 Endemic Equilibrium Point (EE)

The endemic equilibrium point $\mathcal{E}^* = (S^*, E^*, I^*, T^*, R^*, Q^*)$ is the nontrivial equilibrium point in which Typhoid fever disease and Salmonella Typhi bacteria persists in both the human population and environment. To solve for $\mathcal{E}^*$, we let $\dfrac{dS}{dt} = 0$, $\dfrac{dE}{dt} = 0$, $\dfrac{dI}{dt} = 0$, $\dfrac{dT}{dt} = 0$, $\dfrac{dR}{dt} = 0$ and $\dfrac{dQ}{dt} = 0$. Solving for these set of equations can be explicitly expressed in terms of the force of infection Ψ_H. Hence, we derive the equilibrium solutions of different states as

$$\begin{aligned}
S^* &= \frac{\Delta(\eta+\xi)(\theta+\xi)(\sigma+\xi)(\rho+\sigma+\xi)(\Psi_H+\xi)}{-\eta\theta\rho\sigma\Psi_H + (\eta+\xi)(\theta+\xi)(\sigma+\xi)(\rho+\sigma+\xi)(\Psi_H+\xi)^2},\\
E^* &= \frac{\Delta(\eta+\xi)(\theta+\xi)(\xi+\rho+\sigma)\Psi_H}{-\eta\theta\rho\sigma\Psi_H + (\eta+\xi)(\theta+\xi)(\xi+\sigma)(\xi+\rho+\sigma)(\xi+\Psi_H)},\\
I^* &= \frac{\Delta(\eta+\xi)(\theta+\xi)\sigma\Psi_H}{-\eta\theta\rho\sigma\Psi_H + (\eta+\xi)(\theta+\xi)(\xi+\sigma)(\xi+\rho+\sigma)(\xi+\Psi_H)},\\
T^* &= \frac{\Delta\rho\sigma\Psi_H(\theta+\xi)}{-\eta\theta\rho\sigma\Psi_H + (\eta+\xi)(\theta+\xi)(\xi+\sigma)(\xi+\rho+\sigma)(\xi+\Psi_H)},\\
R^* &= \frac{\Delta\eta\rho\sigma\Psi_H}{\eta\theta\rho\sigma\Psi_H + (\eta+\xi)(\theta+\xi)(\xi+\sigma)(\xi+\rho+\sigma)(\xi+\Psi_H)},\\
Q^* &= \frac{C\left(-d+z+\sqrt{\dfrac{((d-z)^2 + (4z\Delta(\eta+\xi)(\theta+\xi)\sigma\phi\Psi_H)}{(C(\eta\xi\Phi + \eta(\xi(\xi+\sigma)(\xi+\rho+\sigma) + \theta(\xi(\xi+\rho)+2\xi\sigma+\sigma^2))\Psi_H + \xi\Phi(\xi+\Psi_H)))}}\right.}{2z},
\end{aligned} \tag{19}$$

where

$$\Phi = (\theta+\xi)(\xi+\sigma)(\xi+\rho+\sigma).$$

1.4.1 Local Stability of EE

Theorem 5. The endemic equilibrium point of system (1) is locally asymptotically stable if $R_0 > 1$, and unstable if $R_0 \leq 1$.

Proof. To determine the local stability of the endemic equilibrium, we let

$$a_1 = \sigma+\xi, \quad a_2 = \delta+\rho+\xi, a_3 = \eta+\xi, \quad a_4 \quad = \theta+\xi, \quad c = d-z.$$

System (1) always has a solution of the form $\mathcal{E}_0$, thus, we proceed by simplifying the system using variable elimination. Specifically, we use the relations,

$$I^* = \frac{\sigma E^*}{a_2}, \quad T^* = \frac{\rho I^*}{a_3}, \quad R^* = \frac{\eta T^*}{a_4}.$$

System (1) is reduced to

$$0 = \Delta + \gamma I - (\Psi + \xi)S, \tag{20}$$
$$0 = \Psi S - \kappa I, \tag{21}$$
$$0 = \phi I - cQ - \frac{zQ^2}{C}, \tag{22}$$

where:

$$\gamma = \frac{\theta\rho\eta}{a_3 a_4}, \quad \kappa = \frac{a_1 a_2}{\sigma}.$$

We solve for S in Equation (20) and substitute it Equation (21). This results in a quadratic equation in I, which we multiply by $(Q + \alpha_0)(1 + \alpha_1 I)$ to obtain the following polynomial;

$$(\Psi + \xi)(\Delta + \gamma I)(Q + \alpha_0)(1 + \alpha_1 I) = \kappa I \Psi (Q + \alpha_0)(1 + \alpha_1 I). \tag{23}$$

The solutions of (23) are substituted into Equation (22) yielding a quartic equation of the form:

$$P_4(Q) = A_4 Q^4 + A_3 Q^3 + A_2 Q^2 + A_1 Q + A_0 = 0.$$

The coefficients of this quartic equation are given by:

$$\begin{aligned}
A_4 &= z^2\sigma\left[\alpha_1\kappa\xi + \beta_H(\kappa-\gamma) + \alpha_1\beta_Q(\kappa-\gamma)\right],\\
A_3 &= z\sigma\left[2C\alpha_1 c\kappa\xi + \alpha_0\alpha_1\kappa\xi z + 2Cc\beta_H(\kappa-\gamma)\right.\\
&\quad \left.+\alpha_0 z\beta_H(\kappa-\gamma) + 2C\alpha_1 c\beta_Q(\kappa-\gamma)\right],\\
A_2 &= C\sigma\left[C\alpha_1 c^2\kappa\xi + 2\alpha_0\alpha_1 c\kappa\xi z + Cc^2\beta_H(\kappa-\gamma)\right.\\
&\quad +C\alpha_1 c^2\beta_Q(\kappa-\gamma) - \Delta\alpha_1\phi z\beta_Q + 2\alpha_0 cz\beta_H(\kappa-\gamma)\\
&\quad \left.-\Delta\phi z\beta_H + \phi z\kappa\xi + \phi z\beta_Q(\kappa-\gamma)\right],\\
A_1 &= C\sigma\left[C\alpha_0\alpha_1 c^2\kappa\xi - C\Delta c\phi\beta_H - C\Delta\alpha_1 c\phi\beta_Q + C\alpha_0 c^2\beta_H(\kappa-\gamma)\right.\\
&\quad \left.+Cc\kappa\phi\xi + Cc\phi\beta_Q(\kappa-\gamma) - \Delta\alpha_0\phi z\beta_H + \alpha_0\kappa\phi\xi z\right],\\
A_0 &= C^2\phi\sigma\left[\alpha_0 c\kappa\xi - \Delta(\beta_H\alpha_0 c + \beta_Q\phi)\right].
\end{aligned}$$

Recall that the basic reproduction number, R_0, is expressed as:

$$R_0 = \frac{\Delta}{\xi}\frac{1}{\kappa}\left[\beta_H + \frac{\beta_Q\phi}{\alpha_0 c}\right].$$

A straightforward algebraic calculation of A_0 gives:

$$A_0 = C^2\phi\kappa\sigma\alpha_0 c\xi\left(1 - R_0\right).$$

Thus, A_0 is positive for $R_0 < 1$ and negative for $R_0 > 1$. Next, we show that $\kappa - \gamma > 0$. From the definitions of a_1, a_2, a_3, and a_4, we have:

$$\frac{\gamma}{\kappa} = \frac{\sigma}{a_1}\cdot\frac{\rho}{a_2}\cdot\frac{\eta}{a_3}\cdot\frac{\theta}{a_4} < 1,$$

which implies that $A_4 > 0$ and $A_3 > 0$. Additionally, the terms involving $(\kappa - \gamma)$ in A_2 and A_1 are strictly positive. Therefore, for $R_0 \geq 1$, all terms in the quartic equation are positive, and $A_0 < 0$ for $R_0 > 1$. Since $P_4'(Q)$ is strictly increasing for $Q \geq 0$, the equation $P_4(Q) = 0$ has a unique positive root $Q^* > 0$ for $R_0 > 1$. For $R_0 = 1$, we have $A_0 = 0$, and the only solution is $Q^* = 0$.

□

1.4.2 Global Stability of EE

To prove the global stability of the EE, we use the Lyapunov concept in [4].

Theorem 6. The EE denoted by $\mathcal{E}^*$ is globally asymptotically stable if $R_0 > 1$, otherwise unstable.

Proof. We define the Lyapunov function as

$$V(X) = \sum_{x\in\{S,E,I,T,R\}} \left(x - x^* - x^* \ln\left(\frac{x}{x^*}\right)\right) + c\left(Q - Q^* - Q^* \ln\left(\frac{Q}{Q^*}\right)\right),$$

where $c > 0$ is a constant to be determined. Each scalar term is nonnegative for positive arguments and equals zero if and only if the variable equals its equilibrium component. Next, we differentiate along trajectories, thus,

$$\dot{V} = \sum_{x\in\{S,E,I,T,R\}} \left(1 - \frac{x^*}{x}\right)\dot{x} + c\left(1 - \frac{Q^*}{Q}\right)\dot{Q}.$$

Substitute the right-hand sides from system (1) and then add and subtract equilibrium terms, we obtain;

$$\begin{aligned}
\left(1-\frac{S^*}{S}\right)\dot{S} &= \left(1-\frac{S^*}{S}\right)(\Delta + \theta R - \Psi_H S - \xi S) = \left(1-\frac{S^*}{S}\right)(\Delta + \theta R^* - \Psi_H^* S^* - \xi S^*) + \theta(R - R^*) \\
&\quad - (\Psi_H S - \Psi_H^* S^*) - \xi(S - S^*), \\
\left(1-\frac{E^*}{E}\right)\dot{E} &= \left(1-\frac{E^*}{E}\right)(\Psi_H S - (\xi+\sigma)E) = \left(1-\frac{E^*}{E}\right)\left[(\Psi_H S - \Psi_H^* S^*) - (\xi+\sigma)(E - E^*)\right], \\
\left(1-\frac{I^*}{I}\right)\dot{I} &= \left(1-\frac{I^*}{I}\right)(\sigma E - (\xi+\delta+\rho)I) = \left(1-\frac{I^*}{I}\right)\left[\sigma(E - E^*) - (\xi+\delta+\rho)(I - I^*)\right], \\
\left(1-\frac{T^*}{T}\right)\dot{T} &= \left(1-\frac{T^*}{T}\right)(\rho I - (\xi+\eta)T) = \left(1-\frac{T^*}{T}\right)\left[\rho(I - I^*) - (\xi+\eta)(T - T^*)\right], \\
\left(1-\frac{R^*}{R}\right)\dot{R} &= \left(1-\frac{R^*}{R}\right)(\eta T - (\xi+\theta)R) = \left(1-\frac{R^*}{R}\right)\left[\eta(T - T^*) - (\xi+\theta)(R - R^*)\right], \\
c\left(1-\frac{Q^*}{Q}\right)\dot{Q} &= c\left(1-\frac{Q^*}{Q}\right)(\lambda_Q(Q) - \lambda_Q(Q^*) + \phi(I - I^*) - d(Q - Q^*)),
\end{aligned}$$

The difference $\lambda_Q(Q) - \lambda_Q(Q^*)$ simplifies algebraically to:

$$\lambda_Q(Q) - \lambda_Q(Q^*) = z(Q - Q^*) - \frac{z}{C}(Q^2 - Q^{*2}) = (Q - Q^*)z\left(1 - \frac{Q + Q^*}{C}\right).$$

We group the five linear-chain terms and Q-term to obtain:

$$\begin{aligned}
\dot{V} = &\left(1-\frac{S^*}{S}\right)\left[\theta(R-R^*) - (\Psi_H S - \Psi_H^* S^*) - \xi(S - S^*)\right] + \left(1-\frac{E^*}{E}\right)\left[(\Psi_H S - \Psi_H^* S^*) - (\xi+\sigma)(E - E^*)\right] \\
&+ \left(1-\frac{I^*}{I}\right)\left[\sigma(E - E^*) - (\xi+\delta+\rho)(I - I^*)\right] + \left(1-\frac{T^*}{T}\right)\left[\rho(I - I^*) - (\xi+\eta)(T - T^*)\right] \\
&+ \left(1-\frac{R^*}{R}\right)\left[\eta(T - T^*) - (\xi+\theta)(R - R^*)\right] + c\left(1-\frac{Q^*}{Q}\right)\left[(Q - Q^*)z\left(1 - \frac{Q+Q^*}{C}\right) + \phi(I - I^*) - d(Q - Q^*)\right].
\end{aligned}$$

Afterwards, we reorganize the terms by grouping the differences $(\Psi_H S - \Psi_H^* S^*)$, $(E - E^*)$, $(I - I^*)$, $(T - T^*)$, $(R - R^*)$, and $(Q - Q^*)$. This gives:

$$\begin{aligned}
\dot{V} = &(\Psi_H S - \Psi_H^* S^*)\left(\frac{S^*}{S} - \frac{E^*}{E}\right) + (\xi(S - S^*)) + ((\xi+\sigma)(E - E^*)) + ((\xi+\delta+\rho)(I - I^*)) \\
&+ ((\xi+\eta)(T - T^*)) + ((\xi+\theta)(R - R^*)) + \left(c\left(1 - \frac{Q^*}{Q}\right)\left((Q - Q^*)z\left(1 - \frac{Q+Q^*}{C}\right) + \phi(I - I^*) - d(Q - Q^*)\right)\right).
\end{aligned}$$

The contribution from each group can now be shown to be nonnegative. For the Q-term, we choose a sufficient small value for c, such that it contributes nonpositively, leading to the desired result. Thus, we conclude that:

$$\dot{V} \le -\xi S^* g\left(\frac{S}{S^*}\right) - (\xi+\sigma)E^* g\left(\frac{E}{E^*}\right) - (\xi+\delta+\rho)I^* g\left(\frac{I}{I^*}\right) - (\xi+\eta)T^* g\left(\frac{T}{T^*}\right)$$

$$-(\xi+\theta)R^*g\left(\frac{R}{R^*}\right)-c_1H(Q,I),$$

where $c_1 > 0$ and $H(Q, I) \geq 0$ is a continuous function vanishing only at $(Q, I) = (Q^*, I^*)$. Since $\dot{V} \leq 0$ for all positive states, and $\dot{V} = 0$ only at the equilibrium X^*, LaSalle's invariance principle implies that every trajectory converges to the equilibrium X^*. Thus, the endemic equilibrium is globally asymptotically stable. □

References

[1] Van den Driessche, P., Watmough, J.: Reproduction numbers and sub-threshold endemic equilibria for compartmental models of disease transmission. Mathematical biosciences **180**(1-2), 29–48 (2002)

[2] Gervas, H.E., Opoku, N.K.-D.O., Ibrahim, S., et al.: Mathematical modelling of human african trypanosomiasis using control measures. Computational and Mathematical Methods in Medicine **2018** (2018)

[3] La Salle, J.P.: The Stability of Dynamical Systems. SIAM, ??? (1976)

[4] Martcheva, M.: Analysis of complex ode epidemic models: Global stability. In: An Introduction to Mathematical Epidemiology, pp. 149–181. Springer, ??? (2015)
